# Mesenchymal stem cell transplantation plays a role in relieving cancer pain

**DOI:** 10.3389/fphar.2024.1483716

**Published:** 2024-11-29

**Authors:** Wen-Jun Zhang, Dingyi Chen

**Affiliations:** ^1^ Department of Rehabilitation Medicine, The Second Affiliated Hospital, Nanchang University, Jiangxi Medical college, Nanchang, China; ^2^ Emergency department, The Second Affiliated Hospital, Nanchang University, Jiangxi Medical college, Nanchang, China

**Keywords:** cancer pain, mesenchymal stem cells, nerve injury, tumor, treatment

## Abstract

Tumors can invade, compress, and damage nerves, leading to persistent pain and seriously affecting the quality of life of patients. However, their treatment is challenging. Sensitization of peripheral receptors, abnormal activity of primary sensory neurons, activation of glial cells, enhanced inflammatory responses, and sensory information transmission contribute towards cancer pain. Therefore, considerable attention has been paid to exploring prospective methods to inhibit the occurrence of these factors and relieve cancer pain. Studies on different types of pains have revealed that the transplantation of functionally active cells into the host has the pharmacological effect of producing analgesia. Mesenchymal stem cells (MSCs) can act as small active pumps to reduce the expression of pain-related molecules and produce analgesic effects. Moreover, MSCs can establish complex communication networks with non-tumor and cancer cells in the microenvironment, interact with each other, and can be used as destinations for inflammation and tumor sites, affecting their potential for invasion and metastasis. This emphasizes the key role of MSCs in cancer and pain management. The pain relief mechanisms of MSCs include neuronutrition, neural protection, neural network reconstruction, immune regulation, and improvement of the inflammatory microenvironment around the nerve injury. All of these are beneficial for the recovery of injured or stimulated nerves and the reconstruction of neural function, and play a role in relieving pain. The pain treatment strategy of cell transplantation is to repair injured nerves and produce analgesic pharmacological properties that are different from those of painkillers and other physiotherapies. Although the therapeutic role of MSCs in cancer and pain is in its early stages, the therapeutic value of MSCs for cancer pain has great prospects. Therefore, in this study, we explored the possible mechanism between MSCs and cancer pain, the potential therapeutic role of therapeutic cells in cancer pain, and some problems and challenges.

## 1 Introduction

Pain can be divided into acute and chronic pain based to time; cancer-related pain is a type of chronic pain (Kuner and Kuner, 2021). The occurrence and development of tumors, which can invade and destroy the surrounding tissue and metastasize along the nerve, leads to the sensitization of peripheral receptors, enhancement of sensory information transmission, and induction of pain. This form of pain is more complex and intense than other types of pain and can be characterized by one or more sensory abnormalities, such as high or low sensory sensitivity, abnormal or dull sensation, hyperalgesia, and spontaneous pain, causing serious psychosomatic damage to patient (Hu and Zhang, 2023; O'Hagan and Mercadante, 2018; Inoue, 2021). Half of the cancer patients can experience pain, and especially in those with advanced or distant metastasis, the incidence of pain is significantly increased and strongly unbearable. In addition to the pain caused by the growth and metastasis of the tumor, pain can also be induced during tumor treatment. Clinically, commonly it can lead to peripheral neuropathy and pathological pain in the process of tumor radiotherapy and chemotherapy (Hu and Zhang, 2023; Zhang et al., 2020a). The occurrence of cancer pain brings great negative emotions to patients, which undermines their confidence and resistance to the treatment of primary diseases (tumors), resulting in a significant decline in their quality of life and even the effect of treatment. Therefore, treatment of cancer pain is very important. Pain relief can improve a patient’s resistance to tumors and provide strong confidence in overcoming the disease. It is worth noting that due to different sources of cancer, different location and depth of tumor invasion, different tumor invasion of nerves, different personal tolerance, different age and psychological tolerance, and other factors, the treatment of cancer pain is relatively difficult; therefore, individualized treatment is carried out according to the different conditions of patients (Hu and Zhang, 2023).

Painkillers are used to relieve pain. They are economically feasible and they achieve therapeutic effects. However, they have drawbacks, such as side effects, long-term dependence, and truncation phenomena, and the therapeutic effect gradually decreases with increasing drug usage. Most importantly, drugs cannot restore damaged or stimulated neurological functions. Nerve reconstruction is the preferred method for nerve repair in surgery; however, it has certain shortcomings such as a large degree of injury or the inability to perform end-to-end nerve anastomosis (O'Hagan and Mercadante, 2018). Our growing understanding of neurophysiology and nerve regeneration far exceeds the ability to surgically rebuild damaged nerves and repair their functions. Autologous nerve transplantation is considered the gold standard for the treatment of peripheral nerve injuries (Inoue, 2021). However, it has inherent defects, such as limited donor nerves, neuroma formation, and pain, with less than half of the patients with autologous nerve transplantation not receiving effective functional repair (Zhang et al., 2020a). Tissue engineering substitutes are used as alternatives to autologous nerve transplantation, and various cells and neurotrophic factors are introduced into these grafts for improvement. However, it is still difficult to obtain satisfactory clinical results. This may be related to the lack of cells to repair damaged nerves, inflammatory responses, injury scar formation, tissue fibrosis, and loss of neurotrophic factors (Philips et al., 2018). Therefore, exploring and identifying promising treatment methods is of great value.

Recently, cell transplantation has entered the field of vision. Researchers have transplanted functionally active cells into the host to increase the pain threshold and exert pharmacological properties of analgesia (Chen et al., 2013). Compared with the above treatment methods, stem cells have great advantages. MSCs have the potential for differentiation and self-renewal and have low immunogenicity. Mesenchymal stem cell transplantation repairs damaged nerves and rebuilds neural networks. They also secrete neurotrophic factors for long-term treatment and pain relief. MSCs are widely used in regenerative medicine, including pain treatment (Wen et al., 2022; He et al., 2020). The anti-inflammatory protein TSG-6 secreted by bone marrow mesenchymal stem cells alleviates neuropathic pain by inhibiting TLR2/MyD88/NF-κB signal pathway in spinal microglia (Yang et al., 2020a). Therefore, there is a clear understanding of the role of MSCs in cancer-related pain. MSCs can establish complex communication networks with non-tumor and cancer cells in the microenvironment, interact with each other, and affect their potential for invasion and metastasis (Lan et al., 2021; Tu and Karnoub, 2022). MSCs and tumor cells have a two-way tendency to guide tumor homing, which can also induce or aggravate pain, an important factor in cancer pain. Moreover, MSCs can relieve pain by reducing the expression of pain-related molecules such as receptors, BDNF, and TLRs. Although the application of MSCs in cancer pain is in its infancy, they can broaden new horizons for the treatment of cancer pain. Therefore, in this study, we discuss the potential relationship between MSCs and cancer pain, their potential application as a treatment for cancer pain, and the problems that need to be resolved for the treatment of cancer pain.

## 2 Brief introduction of MSCs

MSCs are derived from mesenchymal cells and possess stem cell characteristics. The most immature MSCs (true stem cells) can differentiate into other embryonic lineages (Pittenger et al., 1999). MSCs have a variety of unique characteristics, including differentiation potential, colony formation ability, and self-renewal ability. MSCs can differentiate into mesenchymal lineages, such as osteoblasts, chondrocytes, adipocytes, endothelial cells, and cardiomyocytes, and non-mesenchymal lineages, such as hepatocytes and neurons (Lazennec and Jorgensen, 2008; Smirnov et al., 2007; Crapnell et al., 2013). MSCs exist in the adult bone marrow and various tissues, such as adipose tissue, nerve tissue, umbilical cord blood, and dermis (Dai et al., 2007; Jones et al., 2002; Li and Hua, 2017). MSCS can be obtained from different tissues, such as bone marrow, adipose tissue, placenta, or umbilical cord, and can proliferate to produce cellular products for treatment (El Omar et al., 2014). MSCS were cultured as adherent cells *in vitro*, expressing CD105, CD73 and CD90 markers and hematopoietic surface markers CD45, CD34 and CD14, but not expressing CD11b and CD79α or CD19 and HLADR (Dominici et al., 2006).

Moreover, MSCs can exert immunomodulatory and anti-inflammatory effects by interacting with immune cells and through paracrine signaling (Li and Hua, 2017; Lo Sicco et al., 2017). Exosomes secreted by MSCs through paracrine signaling not only have the same effect as MSCs but also function in the targeted delivery of drugs and molecular substances to target organs (Tang et al., 2021). MSCS can also participate in angiogenesis, repair damaged tissues, and maintain tissue characteristics (Bagno et al., 2018). Another important feature of MSCs is their low number of immune foci. On the one hand, MSCs do not express human leukocyte antigen (HLA) II molecules and can hardly induce allogeneic lymphocyte proliferation (Le Blanc and Ringdén, 2005). In contrast, bone marrow-derived MSCs appear to exert immunosuppressive effects *in vitro*. They inhibit the proliferation of T cells in response to alloantigens and mitogens and prevent the development of cytotoxic T cells. MSCs can prolong the survival of skin allografts *in vivo* and exert various immunomodulatory effects (Le Blanc and Ringdén, 2007). This is precisely because of their self-renewal ability, pluripotency, and low immunogenicity, which have become the focus of cell therapy in regenerative medicine and the main source of cells for damaged tissue replacement.

MSCs are the most mature cells used for tissue engineering and regeneration. However, the availability of autologous MSCs is limited. Initially, somatic differentiation was thought to be a stable and irreversible process, but this concept has been disregarded by an increasing number of studies. Somatic cells can be induced to differentiate into stem cells using a variety of methods, including nuclear transplantation, cell fusion, epigenetic modifications, and ectopic gene expression. iPSCs can be induced by upregulating pluripotency-related genes that provide the basis for cell transplantation (Matoba and Zhang, 2018; Eilertsen et al., 2007; Zhang et al., 2023). In 2007, somatic reprogramming of iPSCs was achieved by the transient forced expression of foreign transcription factors in the human body (Teshigawara et al., 2017). Different studies have revealed that MSCs can be obtained from autologous cells by reprogramming and have characteristics similar to those of endogenous MSCs, including pluripotent differentiation and self-renewal (Prakash et al., 2023). Both early stage MSCs and late-stage MSCs induced by iPSCs maintained their multidirectional differentiation potential, showed self-renewal ability without tumorigenesis, and the regenerated bone tissue was helpful in repairing the defective area (Sheyn et al., 2016). It has been found that anti-BMP2 Ab/BMP2 immune complex can promote osteogenic differentiation of iPSCs-derived MSCs (Wu et al., 2018). MSCs induced by iPSCs express typical MSCs surface markers that can differentiate into osteogenic, adipogenic, and chondrogenic cell lines and play a paracrine function (Spitzhorn et al., 2019). Regardless of donor age and cell type, MSCs induced by iPSCs acquire genetic characteristics related to rejuvenation (Spitzhorn et al., 2019). It has been found that MSCs induced by iPSCs have high proliferative activity and differentiation potential and can increase the secretion of paracrine cytokines and growth factors, which are related to transcription and proteomics, but not to differences in individual donors (Lee et al., 2023).

However, some studies have shown that there are significant differences in the three-line differentiation potential between iPSC-derived MSCs and primary MSCs. iPSCs induced the expression of typical MSCs markers, such as high levels of CD73, CD90, and CD105, but this was not enough to distinguish MSCs from other mesodermal precursor cells. iPSC-derived MSCs cannot be used as a substitute for primary MSCs isolated from the bone marrow (Lee et al., 2023). In short, iPSC-induced MSCs are similar to MSCs in differentiation and function, and can bypass the shortcomings related to the use of human-derived MSCs, which makes it possible for MSCs to be used in related diseases.

## 3 MSCs and repair of peripheral nerve injury

**FIGURE 1:**
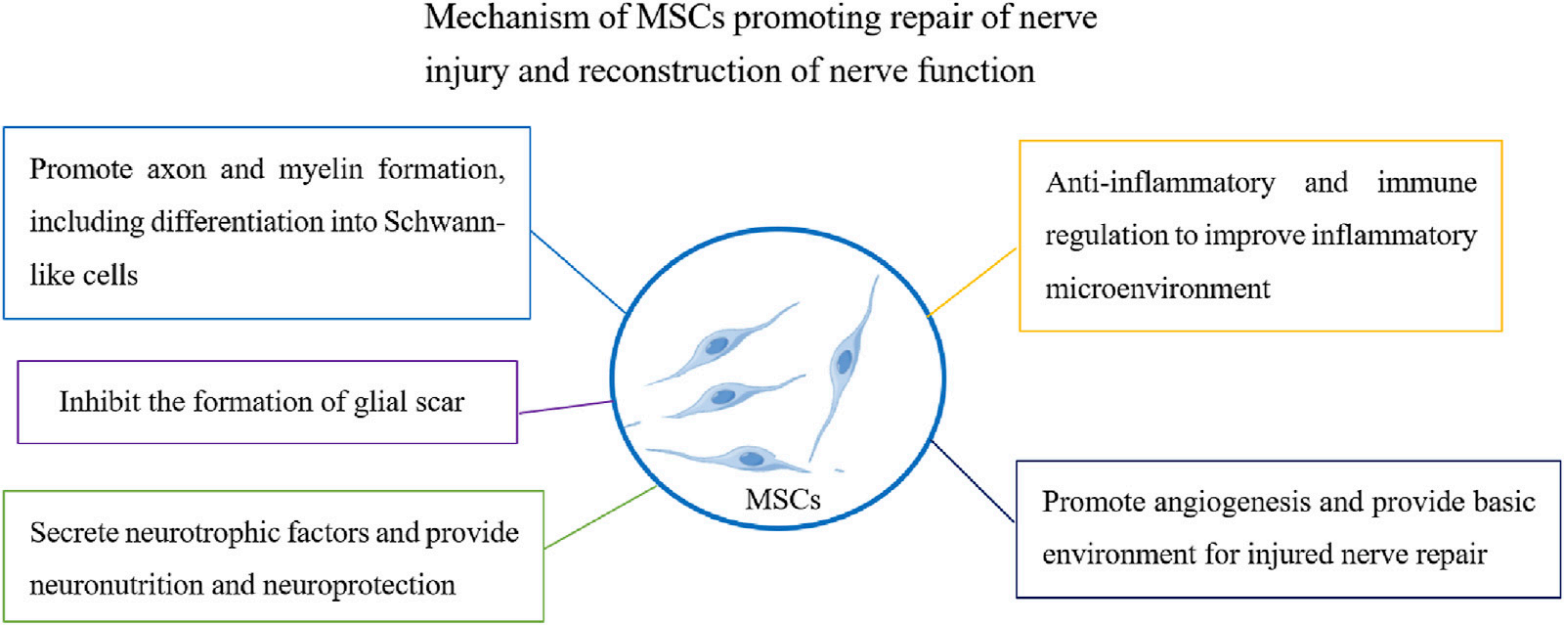
Functional effect of MSCs on promoting regeneration of peripheral nerve injury.

Peripheral nerve injury can be caused by a variety of factors such as trauma, inflammation, tumor invasion, and destruction, leading to sensory and motor dysfunction (Lavorato et al., 2021; Zhang et al., 2020b). Therefore, the repair and functional reconstruction of peripheral nerve injuries are very important. At present, an increasing number of studies have found that the transplantation of functionally active cells (such as olfactory ensheathing cells, neural stem cells, and Schwann cells) can repair injured nerves and the accompanying pain (You et al., 2011; Lee et al., 2021). Cell therapy is one of the most innovative treatments for nerve repair. MSCs are widely favored for applications in regenerative medicine because of their unique characteristics and low immunogenicity. MSCs can support the regeneration of damaged tissues through targeted differentiation. MSCs secrete a wide range of neuroregulatory factors (such as growth factors and cytokines), promote angiogenesis and neuronal survival, inhibit glial scar formation, improve the nerve injury inflammatory microenvironment and immune regulation, and promote nerve injury repair (Madrigal et al., 2014; Teixeira et al., 2013; Mead and Tomarev, 2017) (Figure 1). These functions play important roles in repairing injured nerves, rebuilding nerve function, and relieving pain induced by nerve injury.

Good progress has been made in understanding the therapeutic effects of MSCs on peripheral nervous system injuries. The most important aspect of the regeneration of peripheral nerve injuries is connecting the regenerated axons through the injured area with the distant target nerve to form a new neural network. The transplantation of MSCs to injured sites can replace injured tissue cells, promote axon regeneration and myelin formation, guide axons to distant injured sites, and play a role in nerve bridging (Mathot et al., 2019; Qiu et al., 2015). Studies have shown that MSCs can significantly increase the number of axons after sciatic nerve injury, improve recovery speed and amplitude of muscle action potentials, and promote nerve regeneration (Cooney et al., 2016; Ülger et al., 2021).

It is understood that Schwann cells are the myelin sheath of the peripheral nerve and have the function of protecting and nourishing neurons. Following nerve injury, it can alter the repair phenotype and promote axonal regeneration and remyelination (Jessen and Mirsky, 2016). Another important function of MSCs in repairing injured nerves is differentiation into Schwann-like cells and promotion of injury repair (Peng et al., 2011). The upregulated expression of NGFR, S100B, and KROX20 in Schwann-like cells differentiated from MSCs promotes the formation of neuronal axonal myelin sheaths, regeneration of injured nerves, and improved motor function (Jung et al., 2016). MSCs derived from umbilical cord glia can differentiate into Schwann cell-like cells and promote peripheral nerve regeneration (Choi et al., 2021). Schwann-like cells differentiated from MSCs have better therapeutic effects when they carry nerve growth factors. Other studies have shown that conductive targeted nanofibers can significantly improve the ability of MSCs to differentiate into Schwann cells and promote peripheral nerve axonal regeneration (Hu et al., 2020).

With the development of cell transplantation in nerve regeneration, an increasing number of studies have adopted cells combined with other methods (such as nerve conduits) or carrying specific nerve regeneration factors, which have more advantages for nerve regeneration (Frattini et al., 2012). Transplantation of MSCs carrying dopamine neurotrophic factor (CDNF) promotes nerve regeneration and functional recovery following peripheral nerve injury (Liu et al., 2014). A possible reason for this is that CDNF has significant neuroprotective and neurotrophic effects, and provides a superior environment. The addition of MSCs to nerve conduits can significantly improve nerve regeneration. The mechanism of repairing injured nerves involves establishing a more favorable environment for nerve regeneration by increasing the expression of neurotrophic and angiogenic factor (Shalaby et al., 2017). These studies have revealed the repair function of MSCs in peripheral nerve injury and regeneration, providing a basis and support for the treatment of pain induced by nerve injury.

These studies are based on basic research theories; however, there are few reports of MSCs in clinical trials on the repair of peripheral nerve injury. A recent study reported the case of a 24-year-old male patient who suffered nerve damage in his left forearm due to trauma. The lesion was repaired using a fibular autograft. MSC-derived exosomes (1 mL) containing 5 billion microbubbles were transplanted into the damaged nerve stump. The damaged nerve stump was followed-up for 180 days, during which time neurological examination and electrodiagnostic testing were performed. The results indicated enhanced sensory and motor recovery (Civelek et al., 2024). This study also suggests that MSCs transplantation may have therapeutic effects in clinical trials. However, this study included only one patient, and the number of cases was small, which was not sufficient to confirm the exact effect of the clinical trial treatment of MSCs transplantation. However, further clinical trials and direct evidence are required to confirm this hypothesis. However, MSCs transplantation has potential applications in repairing peripheral nerve injuries.

## 4 MSCs and tumors

**FIGURE 2:**
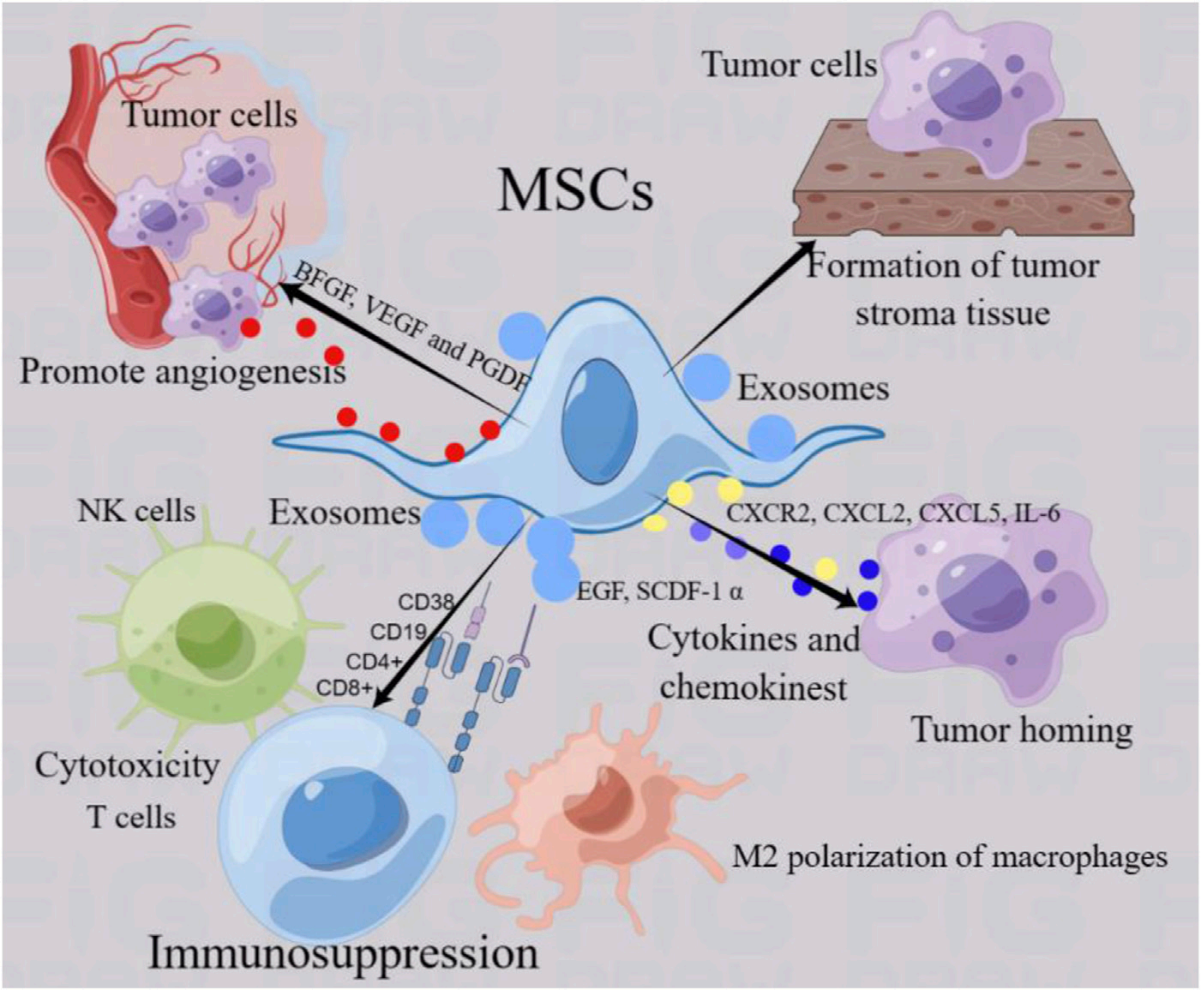
MSCs regulate tumor progression. MSCs can establish complex communication networks with non-tumor cells and cancer cells in the microenvironment and interact with each other, thus regulating the progression of tumors. MSCs function of promoting tumor progression mainly includes the following aspects: 1) MSCs can form tumor-like stromal tissue to provide adhesion and support of tumor cells. 2) MSCs can drive tumor cells to move and induce homing by secreting cytokines and chemokines such as CXCL2 and CXCR2.3) MSCs can regulate immune cells (NK cells, macrophages and T cells) in tumor microenvironment, mediate M2 polarization of macrophages, T cytotoxicity and NK cell killing ability, and mediate immunosuppression, resulting in tumor cell tolerance. 4) MSCs can promote the secretion of endothelial cell and platelet growth factor, promote angiogenesis and provide the basic environment for tumor growth. 5) MSCs produce exosomes through paracrine function, which affect tumor progression by acting on tumor cells and non-tumor cells.

Tumorigenesis is a multifaceted process. Tumor-related stromal cells undergo complex crosstalk with tumor cells, provide appropriate signals, and produce a dynamic extracellular matrix that is conducive to tumor cell invasion and promotes tumor progression (Barcellos-de-Souza et al., 2013; Sato et al., 2004). Tumor formation involves the co-evolution of tumor cells with the extracellular matrix, tumor blood vessels, and immune cells. The successful growth and final metastasis of tumors depend not only on the genetic changes in tumor cells, but also on the adaptive advantages brought about by these mutations in a specific environment (Junttila and de Sauvage, 2013) (Figure 2). The tumor microenvironment consists of non-malignant cells such as endothelial cells, fibroblasts, immune cells, cellular matrix components, and tumor cells. Cellular and acellular components form the tumor matrix, which changes constantly during tumor development and affects tumor growth and chemical resistance (Junttila and de Sauvage, 2013; Xing et al., 2015). MSCs can establish complex communication networks with non-tumor and cancer cells in the microenvironment, interact with each other, and affect the potential for tumor invasion and metastasis (Ferreira et al., 2021). The presence of MSCs in tumor bodies can promote the formation of tumor foci and surrounding matrix-like tissue (Devarasetty et al., 2017). An increasing number of studies have shown that MSCs are involved in different types of tumor growth and metastasis (Barcellos-de-Souza et al., 2013; Devarasetty et al., 2017). These findings revealed a close relationship between MSCs and tumor growth. MSCs promote tumor progression through a variety of mechanisms, including the continuous proliferation of tumor stem cells, inhibition of tumor cell apoptosis, transformation into tumor-associated fibroblasts, promotion of angiogenesis, stimulation of epithelial-mesenchymal transformation, and suppression of the immune response (Barcellos-de-Souza et al., 2013).

One of the important characteristics of MSCs contributing to tumor progression is their bidirectional trend with tumor cells to guide tumor homing. MSCs provide a framework for anchoring tumor cells in the form of a tumor matrix and secrete factors that promote tumor growth (Barcellos-de-Souza et al., 2013). Bone marrow stem cells tend to migrate by establishing a good tumor microenvironment and cytokine network, thus participating in tumor development (Karnoub et al., 2007). Studies have shown that anti-platelet-derived growth factor receptor β (PDGFR β) can seriously hinder the recruitment of bone marrow MSCs to tumor-associated fibroblasts and the attractiveness of tumor cells, and inhibit the homing of MSCs to triple negative breast cancer xenotransplantation (Karnoub et al., 2007). Studies have shown that tumor necrosis factor-α activated CXCR2 ligands (CXCL1, CXCL2 and CXCL5) expressed by MSCs can effectively aggregate and migrate CXCR2+ neutrophils into tumor and significantly promote tumor metastasis (Hernanda et al., 2014). In addition, tumor cells can induce the tendency of MSCs to enter the tumor by releasing driving factors and forming an interaction (Zhang X. et al., 2018). Human bone marrow mesenchymal stem cells were directly into the cerebral hemisphere opposite an established human glioma and showed that MSCs could migrate into xenograft tumor *in vivo,* which may be mediated by platelet-derived growth factor, epidermal growth factor or stromal cell-derived factor-1 α secreted by tumor cells (Cheng et al., 2021). Stereotactic body radiation therapy can promote tumor cells to release stromal cell-derived factor-1 α (SDF-1α) and platelet-derived growth factor-B (PDGFR-B) to bind to the corresponding ligands, resulting in bone marrow mesenchymal stem cells to migrate into the tumor parenchyma. Homing MSCs differentiate into pericytes, induce tumor angiogenesis, and promote tumor regrowth (Razmkhah et al., 2019). Research shows that breast cancer cells can devour MSCs, thereby enhancing their metastasis (Augimeri et al., 2023). Clinical samples from primary aggressive cancers and drug-resistant breast cancer metastases contain a unique population of hybrid cancer cells that co-express pan-cytokeratin and the MSCs marker fibroblast activating protein-a, promoting breast cancer metastasis, chemical resistance and primary tumor formation (Augimeri et al., 2023). Co-culture of MSCs with breast cancer increased oxidative phosphorylation, intracellular ATP, and cancer cell resistance to standard therapies for estrogen receptor-positive (ER+) breast cancer (Buschhaus et al., 2023). Other studies have shown that mesenchymal stem exhibit targeted chemotactic properties for gastric cancer and have effective epirubicin loading and tolerance (Zhou et al., 2024).

MSCs mediate immune responses by inhibiting the activation of natural killer (NK) cells and the activation and basic functions of dendritic cells, regulating the proliferation and function of B cells, and inducing the expansion of regulatory T cells. MSCs regulate the immune response by affecting the activation, maturation, proliferation, differentiation, and effector functions of immune cells, including neutrophils, macrophages, dendritic cells, natural killer cells, and T lymphocytes. Similar effects were observed in MSCs associated with different types of tumors (Castro-Manrreza, 2016). MSCs mediate tumor immunity and inflammation to regulate tumor progression. MSCs produce an immunosuppressive microenvironment by reducing the cytotoxicity of T lymphocytes and natural killer (NK) cells, which differentiate macrophages into the M2 phenotype and reduce the secretion of Th1 cytokines (Castro-Manrreza, 2016). MSCs promote the polarization of macrophages to M2b-like cells and decrease the levels of IL-10 and tumor necrosis factor-α (Kudlik et al., 2016). The expression of indoleamine 2,3-dioxygenase in MSCs significantly reduced the infiltration of CD8 (+) T and B cells and promoted the growth of melanoma and lymphoma (Ling et al., 2014). Studies have shown that endometrial cancer-derived MSCs (EmCaMSCs) isolated from patients express MSCs markers and demonstrate three-lineage differentiation capabilities. EmCaMSCs reduce the expression of CD56, CD4, and CD8, thereby exhibiting immunosuppressive effect (Wang et al., 2022). Recent studies have shown that an engineered mesenchymal stem cell biotherapy platform (MB/IL12-MSC) that combines the immune gene vector IL12 (pIL12) and the photosensitizer methylene blue (MB) limits the expression and distribution of IL12 at tumor sites, thereby minimizing potential toxicity while stimulating sufficient anti-cancer immunity. Immune activation induced by MB/IL12-MSC establishes long-term systemic immune memory to prevent tumor recurrence (Zhang et al., 2022). Other studies have shown that gastric cancer-derived MSCs activate the AKT/c-Myc/mTOR pathway in gastric cell lines and upregulate CD276 expression, thereby promoting the migration of gastric cancer cells (Gao et al., 2023).

In addition, MSCs can secrete growth factors or cytokines, such as vascular endothelial growth factor, platelet-derived growth factor, and basic fibroblast growth factor, which promote angiogenesis and lead to tumor progression and metastasis *in vivo* (Hata et al., 2010). MSCs stimulate angiogenesis via a paracrine mechanism and accelerate the regeneration of ischemic or injured tissues. Tumor necrosis factor-α activated bone marrow mesenchymal stem cells secrete pro-angiogenic cytokines, including IL-6 and IL-8, which increase the homing of endothelial progenitor cells to stimulate angiogenesis (Kwon et al., 2013). Another characteristic of MSCs that promotes tumor progression is the secretion of exosomes in the tumor microenvironment. Exosomes, also known as intracavitary vesicles, are a subtype of extracellular vesicles formed by endophagocytic pathways, are usually 30–150 nm in diameter, and are secreted by all types of cells. Exosomes are widely found in human body fluids, such as plasma, urine, semen, saliva, amniotic fluid, synovial fluid, and tears (Doyle and Wang, 2019). In the tumor microenvironment, exosomes are produced and secreted by cells, including tumor cells and mesenchymal stem cells. Exosomes can act as information substances between tumor cells and MSCs to regulate tumor progression (Liu et al., 2022; de Araujo Farias et al., 2018). MSCs are regarded not only as receivers of tumor signals but also as effective producers of their own exosomes, which transmit information to neighboring cells, reprogram the microenvironment, and provide a favorable environment for tumor survival and expansion (Whiteside, 2018a; Boyiadzis and Whiteside, 2017). It has been found that the exosomes secreted by bone marrow mesenchymal stem cells can be absorbed by breast cancer BM2 cells, which promotes the metastasis of breast cancer cells (Ono et al., 2014). MSCs-derived exosomes promote EMT, invasion and migration of lung cancer cells, while silencing the expression of TGF-β1 in MSCs may inhibit MSCs-derived exosomes-mediated EMT formation of lung cancer by inactivating Smad2/3, Akt/GSK-3β/β-catenin, NF- κB, ERK, JNK and p38MAPK signaling pathways (Zhao et al., 2018).

Exosomes produced by MSCs contain immunosuppressive substances that can inhibit anti-tumor immune responses and promote tumor growth (Roccaro et al., 2013). Exosomes derived from normal bone marrow mesenchymal stromal cells inhibit the growth of multiple myeloma cells. In multiple myeloma, exosomes derived from bone marrow mesenchymal stromal cells have high levels of carcinogenic proteins, cytokines, and adhesion molecules, leading to tumor growth (Roccaro et al., 2013). Moreover, exosomes derived from tumor cells are commonly found in the tumor environment and body fluids of cancer patients (Ludwig et al., 2017; Whiteside, 2018b). Studies have shown that microvesicles derived from adipose tissue-derived human immortal stem cells can be internalized by primary ovarian cancer cells, reducing the metabolic activity of ovarian cancer cells and inducing cancer cell death, resulting in the reduced migration of tumor cells (Szyposzynska et al., 2023). The anti-cancer effect of MSC-derived microvesicles is likely achieved through the delivery of molecules that induce cell cycle arrest and cell death (p21, tumor suppressor p53, executive factor semitranspainase 3) and pro-cell death regulatory factors (bad, BMI, FasL, p27, TRAIL-R1, and TRAIL-R2). Studies have shown that adipose-derived MSCs with low expression of CD90 and their exosomes inhibit tumor growth in tumor-bearing mice. Tumor control is related to the reduced proliferation and migration of tumor cells mediated by adipose-derived MSC-derived exosomes and enhanced apoptosis of tumor cells (Li et al., 2020). Tumor cell-derived exosomes transmit information to non-tumor or malignant cells in the tumor microenvironment, including MSCs. In tumors, tumor-derived exosomes carry a variety of immunosuppressive signals, invalidate anti-tumor immune effector cells, and promote tumors to escape immune control (Li et al., 2020).

However, some studies have obtained contradictory results, suggesting that MSCs have an inhibitory effect on tumors. This phenomenon has been reported in some tumors such as hepatocellular carcinoma (Qiao et al., 2008), breast cancer (Sun et al., 2009), and melanoma (Otsu et al., 2009). Human bone marrow mesenchymal stem cells can inhibit the malignant phenotype of human hepatocellular carcinoma cell lines (H7402 and HepG2) both *in vitro* and *in vivo*, including proliferation, colony-forming ability, and oncogene expression (Lu et al., 2019). Studies have found that direct inoculation of MSCs into subcutaneous melanoma induces apoptosis and inhibits tumor growth (Otsu et al., 2009). Interestingly, in the case of a high dose of MSCs, MSCs have potential cytotoxicity, whereas when the dose of MSCs is reduced, toxicity to tumor cells is reduced (Otsu et al., 2009). This means that the number of MSCs may affect the progression of tumor cells. Therefore, the number of MSCs should be considered in tumor therapy. Bone marrow MSCs promote the death of glioma cells by regulating protein changes related to cell death and cell cycle progression, inhibiting the epidermic-mesenchymal transition and PI3K/AKT pathways (Lu et al., 2019). Moreover, MSCs inhibit tumor growth by inhibiting angiogenesis. Studies have shown that the co-administration of MSCs and glioma cells leads to a significant reduction in tumor volume and vessel density, inhibiting the growth of human glioma cells. It was further shown that in the conditioned medium co-cultured MSCs with glioma, the expression of platelet-derived growth factor (SDF)-BB and leukocyte factor (IL)-1 β was reduced, and the recruitment of endothelial progenitor cells and the ability to form endothelial tubes were significantly impaired, indicating that MSC inhibits tumor angiogenesis by releasing anti-angiogenic factors (Ho et al., 2013). In addition, MSCs exert anti-tumor effects through cell-to-cell contacts. One study found that umbilical cord MSCs inhibit the growth of glioblastoma by interacting directly with glioblastoma cells or indirectly regulating the release of soluble factors (Bajetto et al., 2017). This suggests that this direct cell-to-cell interaction may lead to inhibition of tumor growth. Another possible reason is that phenotypic changes of MSCs in the tumor microenvironment are related (Mantovani and Locati, 2013). With changes in the tumor microenvironment, the phenotype of MSCs can change accordingly, and their effect on tumor cells can also vary, producing inhibitory or promotion functions (pro-inflammatory MSC1 and anti-inflammatory MSC2). The MSC1 phenotype can inhibit tumor colony formation and tumor sphere growth and affect tumor migration and invasion, while MSC2 has the opposite effect (Betancourt, 2013; Waterman et al., 2012). The proliferation and survival of bone marrow mesenchymal stem cells induce a pro-inflammatory phenotype and enhance their killing effect on K562 leukemia cells (Aru et al., 2023).

In summary, MSCs interact with tumor cells and play an important role in regulating tumor progression. Numerous studies have revealed the role of MSCs in promoting tumor growth and metastasis. However, few studies have demonstrated the inhibitory effects of MSCs on tumors. The specific cause and mechanism of this opposite effect are unclear and may be related to the concentration of MSCs, method of experimental design, heterogeneity of MSCs, phenotypic changes in the tumor microenvironment, and microenvironment reprogramming. As key components of the tumor microenvironment, MSCs contribute to the survival and reproduction of tumor cells and inhibit natural anti-tumor immune responses. Furthermore, the reported differences in the ability of MSCs to inhibit or promote cancer development may be due to differences in experimental settings such as animal models, cell lines, doses, and duration of treatment. Therefore, whether MSCs should be considered antitumor drugs or therapeutic targets for cancer treatment remains controversial. Therefore, further *in vivo* studies should be conducted to rigorously assess the tumor-inhibitory effects of MSCs. In addition, there is a need to develop effective methods that can distinguish between anti-tumor mesenchymal stem cells to improve the specificity of targeted therapy. Given the results of the current study, the complex interaction patterns between MSCs and tumors have prompted researchers to use stem cells in anticancer therapies. However, the rapid development of genetic engineering technology has made it possible to use viral vectors, non-viral vectors, and other transfection tools to load drugs with good anti-tumor effects into MSCs. Moreover, the tumor tropism of MSCs allows them to release drugs accurately near the tumor site, which theoretically increases the safety and effectiveness of the treatment. Additionally, a growing research suggests that MSC-derived exosomes can be used as powerful cell-free therapies for cancer. Therefore, MSC-based therapies are becoming attractive options for cancer treatment. MSCs can regulate the immune response to tumor diseases and the localization of tumor sites. The interaction between MSCs and tumors is involved in the induction or inhibition of cancer progression and metastasis. However, the differences in the impact of MSCs on cancer development remain largely unexplored, which has hindered the transition of MSC-based applications from laboratory to clinical use. Therefore, we must fully understand the mechanism of the interaction between MSCs and tumors, and the relationship between MSCs and other molecular substances in the tumor microenvironment. Better control of the function of MSCs in tumor progression could provide support for MSCs as a future tumor treatment.

## 5 MSCs and the possible mechanism of cancer pain

**FIGURE 3:**
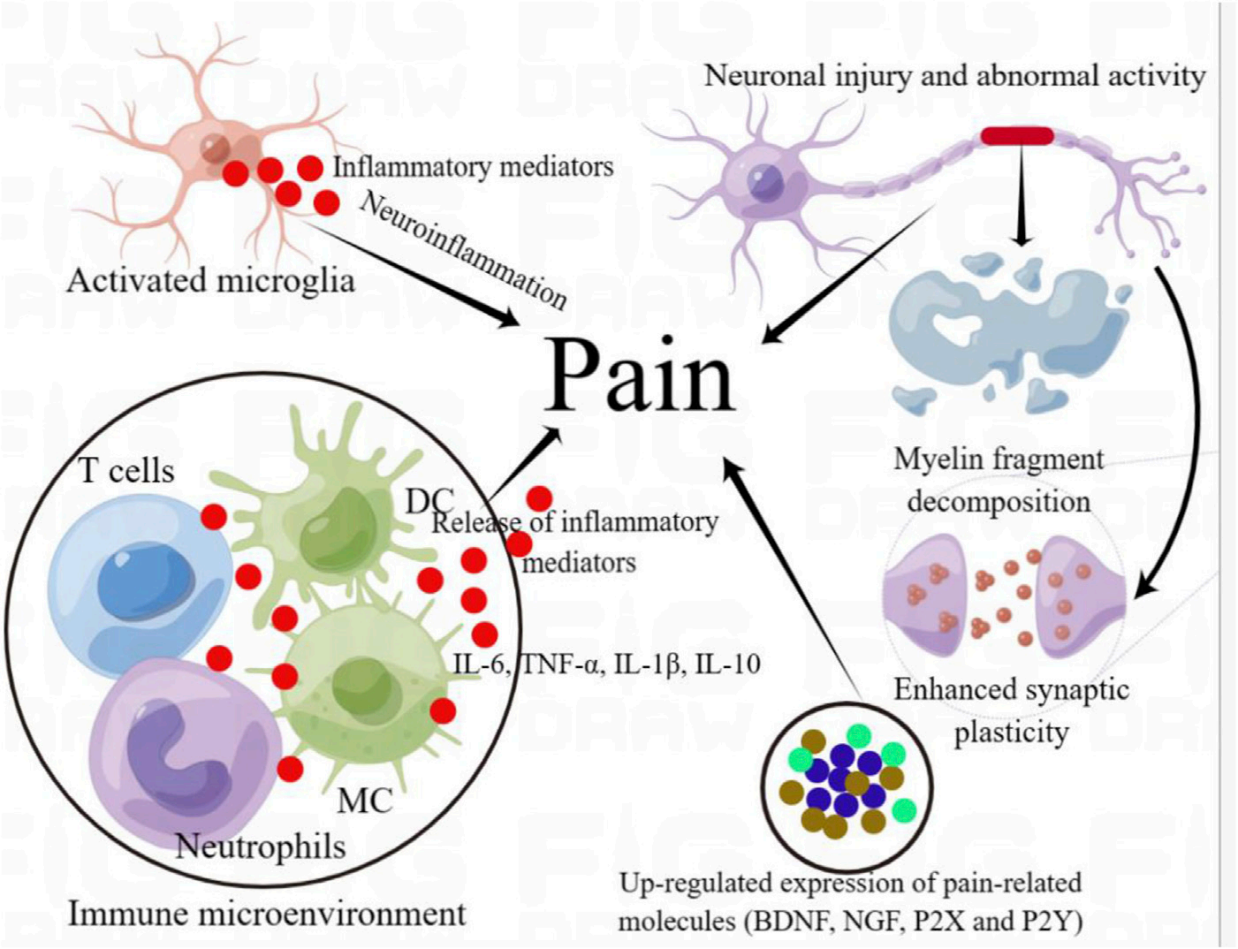
Mechanism diagram of cancer pain. In the process of tumor growth, invasion and metastasis, microglia can be activated, their phenotypes change, turn to pro-inflammatory phenotypes, release inflammatory mediators, and induce neuroinflammation. Moreover, the activity of immune cells in tumor immune microenvironment changed with the progression of tumor, M2 polarization of macrophages and decreased T cell toxicity resulted in immunosuppression, which provided immune escape for tumor cell growth. These changes in cell activity can enlarge nerve tissue injury by releasing a series of inflammatory cytokines, such as IL-6 and IL-1 β. Furthermore, the expression of tumor-related pain molecules (such as BDNF and P2X) can be significantly increased. These changes in tumor progression can lead to neuronal inflammation, neuron injury and abnormal neuronal discharge and enhanced synaptic plasticity, enhance sensory information transmission, lead to central sensitization and produce pain.

Tumor development causes nerve invasion or perineural spreading. Nerve invasion is a histological feature of tumor cell invasion. Perineural tumor spread refers to a naked-eye visible tumor that extends along the primary focus to the nerve, which is defined as more advanced and has clinical features, including pain (Bakst et al., 2019). During tumor development, tumor cells can expand and extend along nerve fibers, which can invade and destroy peripheral nerve tissue and induce pain (Mantyh, 2014). On the one hand, the pain induced by the tumor itself, which can damage and invade the nerves during tumor growth, invasion, and metastasis, leads to the sensitization of peripheral receptors, enhances sensory information transmission, sensitizes the central nervous system, and leads to the generation of pain (Zhang et al., 2020c; Mantyh, 2013) (Figure 3). Most studies have used the bone cancer pain model, one of the most common types of cancer pain, to simulate bone metastasis and destruction, or primary bone tumors in human primary cancer. Different types of tumor cells (such as breast extract and osteosarcoma cells) are injected into the bone marrow cavity or other parts. These tumor cells can settle, grow, destroy bone and peripheral nerves, and cause persistent pathological pain (Zhang et al., 2020c; Shenoy et al., 2016; Huo et al., 2018a). An animal model of bone cancer pain confirmed that tumor cells can stimulate osteoclast activity, leading to osteolysis and destruction, and induce pain (Anisiewicz et al., 2019; Yuan et al., 2021). Studies have shown that p38MAPK (P38) subtype MAPK11 (p38 β) upregulates the expression and secretion of monocyte chemoattractant protein-1 in breast cancer cells and promotes the differentiation and activity of osteoclasts (He et al., 2014). Moreover, we found that the functional connections between the main hubs of the ascending and descending pain pathways in the process of cancer pain, such as the periaqueductal gray matter, amygdala, thalamus, and cortical somatosensory areas, were affected by cancer pain state (Buehlmann et al., 2019). Therefore, the inhibition of pain pathway information or reduction in signal transmission can prevent cancer pain. Cytokines and mediators (such as IL-6 and IL-1 β) released by various cells in the microenvironment may lead to a vicious circle of osteolytic bone metastasis and expand cancer pain (Sun et al., 2017a; Zhao et al., 2013). In addition, the tumor immune microenvironment, involving immune cells such as lymphocytes, macrophages, neutrophils, dendritic cells, inflammatory mediators, and tumor metastasis, constitutes important characteristics of cancer pain (Ma et al., 2023). It has been found that β-endorphin can increase T cell proliferation and NK cell killing activity, increase the relative number of T cell subsets, regulate immune cell function and relieve cancer pain (Du et al., 2016). Second, the pain induced by tumor treatment, including radiotherapy and chemotherapy, causes additional damage to patients rather than to the tumor itself (Autio et al., 2013). Several chemotherapies can damage the peripheral nervous system and cause neuropathies. Chemotherapy-induced peripheral neuropathy can affect all nerve fibers, with sensory neuropathy being the most common. The symptoms include impaired tactile sensitivity, tingling, numbness, sensory abnormalities, sensory disorders and pain (Khasabova et al., 2021). Studies have shown that PECS-101 (former HUF-101), a CBD fluorinated analog can reduce paclitaxel-induced tumor necrosis factor, IL-6 and Aif1 (Iba-1) expression and the loss of nerve fibers in the epidermis, and activate PPARγ in macrophages to prevent mechanical and cold hyperalgesia caused by PTX (Silva et al., 2022).

Many cellular and molecular substances are involved in the process of cancer pain, including glial cell activation, immune cell infiltration and release of inflammatory mediators, abnormal activity and plasticity of sensory neurons, release of neurotransmitters and increased expression of pain-related molecules, which can induce pain. Glial activation and neuroinflammation contribute to cancer pain (Zhu et al., 2015; Tu et al., 2021). Studies have shown that inhibiting astrocyte activation induced by bone cancer pain and reducing the expression of inflammatory mediators and neuroinflammation can relieve cancer pain (Jiang et al., 2017a). Microglia play a key role in cancer-related pain (Jiang et al., 2017b). Microglia exhibit different activation states that participate in the development of pain. In the M1 phenotype, microglia release pro-inflammatory cytokines and neurotoxic molecules, promoting inflammation and cytotoxicity. In contrast, M2 microglia secrete anti-inflammatory cytokines and nutritional factors that promote repair, regeneration, and restoration of function (Tu et al., 2021). In the process of bone cancer pain, spinal microglia showed increased M1 polarization, decreased M2 polarization, increased IL-1β expression and decreased IL-10 expression (Huo et al., 2018b). Dehydrotetrahydropalmatine (10 mg/kg) has an obvious antinociceptive effect accompanied by inhibition of the M1 phenotype of spinal cord microglia and upregulation of the M2 phenotype, which reduces neuroinflammatory reactions and bone cancer pain (Huo et al., 2018b). MSCs inhibit glial cell activation and participate in pain relief. Exosomes derived from MSCs can reduce the activation of microglia, astrocytes, oligodendrocytes and macrophages, reduce the levels of inflammatory mediators IL-1β, IL-6 and IL-8, and relieve pain (Shiue et al., 2019). Bone marrow mesenchymal stem cells can significantly increase the mechanical withdrawal threshold, reduce the contents of IL-1β and tumor necrosis factor-α, inhibit the activation of microglia and reduce mechanical hyperalgesia (Huang et al., 2018).

The abnormal activation and release of neurotransmitters (such as GABA) by sensory neurons enhances the transmission of sensory information and contributes to the occurrence of cancer pain. After the occurrence of pain, non-adaptive molecular changes occur in the cell bodies and axons of primary sensory neurons in the dorsal root ganglion, leading to hypersensitivity and hyperexcitation of sensory neurons (peripheral sensitization) (Berta et al., 2017; Zhu et al., 2017). Neuronal silencing factor (NRSF) knockout attenuates chronic pain rather than acute pain hypersensitivity induced by nerve injury. An siRNA-mediated neuronal resting knockout reverses chronic pain hypersensitivity induced by nerve injury in rats (Zhang J. et al., 2018). Regulation of the spinal cord RhoA/ROCK2 pathway by CXCR4 promotes neuronal sensitization and pain hypersensitivity in bone cancer (Xu et al., 2020). The enhancement of excitatory neurons in the anterior cingulate cortex and direct descending projection fibers from the dorsal corticospinal tract to the contralateral lumbar spinal dorsal horn help maintain mechanical anaphylaxis induced by bone cancer (Chiou et al., 2016). The anterior cingulate cortex may be a potential target for the treatment of cancer pain.

Moreover, abnormal neuronal discharges can trigger the progression of cancer pain by synthesizing and releasing neurotransmitters such as glutamatergic transmitters (Zhou et al., 2017). Studies have shown that downregulation of GABA receptors contributes to the development and maintenance of cancer pain (Zhou et al., 2017). Enhanced synaptic transmission of GABA in VLPAG neurons during bone cancer pain (Takasu et al., 2015). Oxycodone inhibits the enhancement of GABAergic synaptic transmission in VLPAG neurons by activating the KIR 3.1 channel and inhibiting bone cancer pain (Takasu et al., 2015). During tumor progression, nociceptive and non-nociceptive mechanoreceptor neurons exhibit changes in nerve discharge patterns associated with increased nociceptive activity, which increases the release of glutamate transmitters (Zhu YF. et al., 2020). When glutamate is injected into the muscle near the femoral head, both types of mechanoreceptor neurons exhibit excitatory responses, resulting in bone cancer pain (Zhu YF. et al., 2020). This suggests that glutamatergic signal transduction is related to cancer pain and may be a factor in the peripheral sensitization and tactile hypersensitivity induced by bone cancer. MSCs can regulate neuronal activation and neurotransmitter release. MSCs deliver exogenous miRNAs to neurons and induce neuronal differentiation and glutamate transporter expression (Lee et al., 2014). Poly γ-glutamic acid, as an exogenous promoter, promotes the differentiation of human bone marrow stem cells into chondrocytes (Antunes et al., 2015). Human bone marrow stem cells and their derived extracellular vesicles can reduce over-excitation of sensory neurons and relieve pain (Ai et al., 2023). In cancer pain, MSCs can mediate the relief of mechanical pain abnormalities by reducing the mitochondrial bioenergy induced by cisplatin in DRG neurons (Boukelmoune et al., 2021). These studies suggest the contribution of sensory neurons to cancer pain and suggest that targeted MSCs may play a potential role in regulating neuronal activity in cancer pain.

**FIGURE 4:**
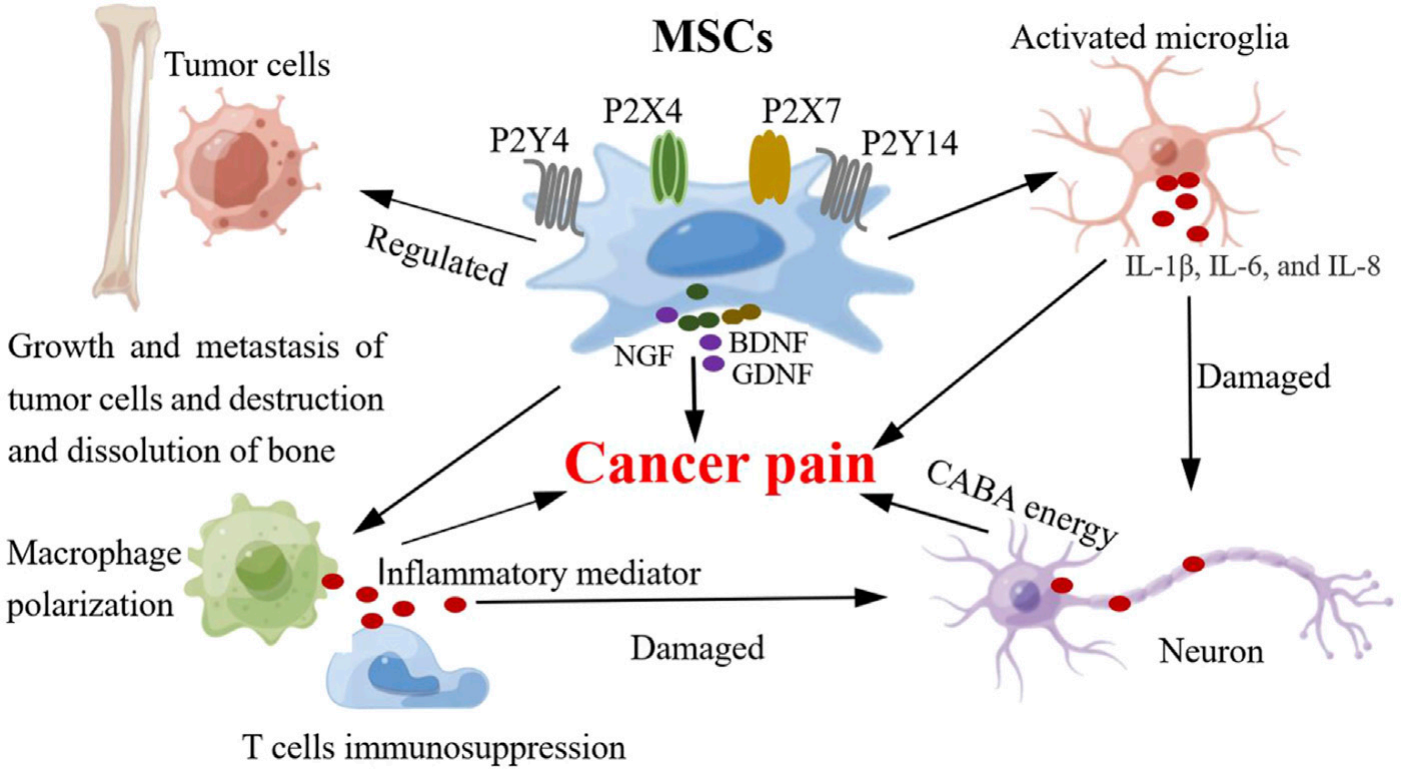
MSCs and the possible mechanism of cancer pain. The involvement of MSCs in the mechanism of cancer pain includes the following functions: 1) MSCs regulate the activity of glial cells (microglia), release pro-inflammatory cytokines (IL-6, IL-8 and IL-1β), which can produce neuroinflammatory response, damage and stimulate neurons, and induce pain. 2) MSCs can regulate neuronal activity and synaptic plasticity, enhance synaptic transmission (GABA neurotransmitter), enhance sensory information transmission, and produce pain. 3) MSCs can regulate the activity of immune cells in inflammatory microenvironment, produce inflammatory mediators, expand inflammatory response, damage nerve tissue and neurons, and induce pain. 4) MSCs can affect bone destruction and dissolution and induce pathological pain by regulating the activity of tumor cells. 5) MSCs can express and secrete some pain-related molecules (NGF, BDNF, P2X and P2Y). The expression of these pain molecules is upregulated and promotes the progression of pain.

MSCs secrete neurotrophic factors that repair injured nerves and promote nerve regeneration. However, oddly enough, some nutritional factors secreted by MSCs (such as NGF, BDNF and GDNF) may be involved in the occurrence of pain as pain-related molecules. Generally, the increased expression of neurotrophic factors in nerve injury is beneficial for axonal regeneration (Terada et al., 2018) (Figure 4). However, neurotrophic factors may play opposing roles as pain signaling molecules in the occurrence of pain (Reis et al., 2022). In a model of pain, the expression of NGF and BDNF in the dorsal root ganglion increased and the mechanical threshold decreased, whereas inhibition of their expression relieved pain (Seki et al., 2018). Other studies have shown that reducing the levels of BDNF, NGF, and GDNF in the dorsal root ganglion helps relieve pain (Cobianchi et al., 2013). These neurotrophic factors also play important roles in cancer-induced pain. In a model of bone cancer pain induced by breast cancer, BDNF mRNA and protein in the L3 dorsal root ganglion were significantly increased and were located in small neurons (mainly nociceptive neurons) rather than in medium or large neurons (non-nociceptive neurons), and the expression level of NGF was also increased (Tomotsuka et al., 2014). It has been suggested that BDNF is a key molecule in bone cancer pain, and the NGF-BDNF cascade reaction may be related to the occurrence of bone cancer pain. Although neurotrophic factors secreted by MSCs promote the regeneration of injured nerves, these factors secreted by MSCs may be used as nociceptive factors to promote the development of pain. In bone cancer pain, short-term exposure of cancer-related fibroblasts, MSCs, and osteoblasts to pH 6.8 can promote the expression of inflammatory and nociceptive mediators (NGF, BDNF, IL-6, IL-8, IL-1b, and CCL5), leading to nociceptor sensitization and hyperalgesia (Di et al., 2017). However, some studies have shown that adipose-derived MSCs combined with pregabalin can significantly increase BDNF expression, promote the recovery of injured neurological function, and relieve neuropathic pain (Yousof et al., 2021). This may be related to the functional role of BDNF in the repair of nerve injuries mediated by MSCs.

MSCs express a variety of receptors that play important roles in their function. Although these molecular substances are not directly released to function outside the cell, they can sense external signals and participate in pain as a medium for pain information transmission. Purinergic receptors are pain-related signaling molecules that play a key role in the occurrence of pain. Purinergic receptors can be divided into P1 and P2 families, and P2 receptors can be divided into P2X and P2Y. P2X receptors are ion channels, whereas P1 and P2Y are G protein-coupled receptors (Patritti-Cram et al., 2021). P2X receptors can be divided into seven subtypes (P2X1, P2X2, P2X3, P2X4, P2X5, P2X6, and P2X7). The P2X receptors are activated by ATP. P2Y receptors contain subtypes P2Y1, P2Y2, P2Y4, P2Y6, P2Y11, P2Y12, P2Y13, and P2Y14, which can be activated by ADP, UTP, UDP, and ATP (Patritti-Cram et al., 2021). The P2X and P2Y receptors play important roles in pain signal transmission. The P2X and P2Y receptors can act as pain-related molecular mediators that contribute to pain progression, including cancer pain (Franceschini and Adinolfi, 2014). There are differences in their expression in the nervous system; for example, P2X2 and P2X3 receptors are expressed in sensory neurons, P2X4 receptors are mainly expressed in microglia, and P2X7 receptors can be expressed in microglia and other cells (astrocytes, oligodendrocytes, neurons, and macrophages). Under physiological conditions, P2X receptors are in a state of low activity; however, when the body is in a pathological condition (tumorigenesis), cells release ATP and activate P2X receptors to participate in tumor progression (Zhang, 2021; Di Virgilio et al., 2021). Generally, P2X receptor activation or overexpression promotes cancer pain progression, whereas inhibition of P2X receptor activation can relieve pain (Zhang et al., 2020d). The expression of the P2X7 receptor increases in bone cancer pain, and the injection of a P2X7 receptor antagonist in the ventrolateral region of the periaqueductal gray could partially antagonize the analgesic effect of tramadol on bone cancer pain in rats (Li et al., 2018).

Recent studies have identified the expression of the P2X and P2Y receptor subtypes in MSCs. P2 receptors regulate MSCs self-renewal and cell fate, and P2X6, P2Y4, and P2Y14 receptors may be key factors in MSC regulation (Zippel et al., 2012). The expression of P2X receptors is regulated by the phenotype of the MSCs that differentiate into specific lineages (Zippel et al., 2012). However, the expression patterns of P2 receptors in MSCs and their fate during differentiation differ (Jiang LH. et al., 2017). It has been found that P2Y4 and P2Y14 receptors are involved in the commitment of early MSCs, and P2Y1 receptor plays a key role in controlling the differentiation of MSCs into endothelial cells or smooth muscle cells (Zhang Y. et al., 2020). This indicates that P2 receptors may control MSC differentiation. This study (Zhang Y. et al., 2020) also revealed that MSCs can regulate pain by mediating P2 receptors. MSC-conditioned medium attenuates mechanical and thermal hyperalgesia in rats with neuropathic pain by inhibiting the upregulation of P2X4 and P2X7 receptors in the spinal cord (Masoodifar et al., 2021). Although there is no direct evidence for the role of MSC-mediated P2 receptors in cancer pain, these studies demonstrate a relationship between MSCs and P2 receptors and their role in pain regulation. This also indicates that P2 receptors can be molecular targets for cancer pain therapy.

TLRs are a type I transmembrane receptor that interacts with adaptive ligand recognition and helper molecules to perform their function (Lacagnina et al., 2018). TLRs are located at the neuroimmune interface, and their activation affects glial cells (including microglia and astrocytes), sensory neurons, and other cell types, thus participating in nociceptive processes and leading to persistent pain (Lacagnina et al., 2018; Thakur et al., 2017). TLRs have been identified in an increasing number of studies and have been shown to play a key role in different types of pain. TLRs also participate in nociceptive signaling and cancer pain (Navia-Pelaez et al., 2023). Cisplatin induces persistent hyperalgesia in wild-type mice, while the pain threshold increases in TLR3 (-/-) and TLR4 (-/-)-deficient mice, which reduces chemotherapy-induced hyperalgesia (Park et al., 2014). In addition, TLRs are associated with the progression of various types of cancers and play regulatory roles in tumor development (Huang et al., 2022; Yu et al., 2014). Anti-hepatoma effects of TLR7 agonists by inhibiting the self-renewal of tumor stem cells (Ren et al., 2016). This indicates a close relationship between TLRs and cancer-related pain. TLRs proteins, such as TLR4, are also expressed in MSCs (Hashemzadeh et al., 2018). Studies have shown that bone marrow mesenchymal stem cells express TLR4 mRNA, and lipopolysaccharide can activate the functional expression of TLR4 in bone marrow mesenchymal stem cells (Shi et al., 2007). TLR4 activation triggers neuroimmune activation and subsequent cytokine expression, whereas TLR4 antisense oligodeoxynucleotides (ODN) can inhibit microglial activation, neuronal hypersensitivity, and reduce hyperalgesia (Tanga et al., 2005). The anti-inflammatory protein TSG-6 secreted by bone marrow mesenchymal stem cells relieves pain by inhibiting TLR2/MyD88/NF-κB signal pathway in spinal microglia and reduces the expression of proinflammatory cytokines IL-1β, IL-6 and TNF-α induced by microglia, which is induced by Pam3CSK4, a specific agonist of TLR2 (Yang et al., 2020b). Other studies have found that the TLR4 ligand lipopolysaccharide protects MSCs from oxidative stress-induced apoptosis and improves their survival rate (Wang et al., 2009). This indicates that there is an interaction between MSCs and TLRs, and that the activation of TLRs can promote the transmission of pain sensory information and the occurrence of pain sensation. Thus, TLRs are potential targets for the treatment of cancer pain.

## 6 Prospect of MSCs and cancer pain treatment

The degree of development and manifestation of cancer pain is related to different tumor types, scope of invasion and metastasis, depth of tumor invasion, degree of nerve stimulation or injury, age, and personal tolerance. There are significant differences in the individual manifestations of pain, which leads to a lack of unified standards for clinical diagnosis and treatment. Therefore, pharmacological methods for the treatment of cancer pain should be individualized according to individual patient conditions. Clinically, different kinds of painkillers (such as opioids, non-opioids and other auxiliary painkillers) are used to relieve pain. Although the use of drugs is economical and feasible and relatively effective, they have some drawbacks such as side effects, dependence, and poor long-term efficacy, which also bring great challenges to the treatment of cancer pain. Importantly, these drugs do not improve nerve injury or stimulation, nor do they restore nerve function. Nerve reconstruction is the first choice for nerve repair in surgery; however, it has certain shortcomings such as a large degree of damage or the inability to perform end-to-end nerve anastomosis. Tissue engineering substitutes are used as alternatives to autologous nerve transplantation; however, it remains difficult to obtain satisfactory clinical results. Other treatment methods include physical therapy and rehabilitation. Although these methods relieve pain to a certain extent, they have short-lived effects and cannot repair damaged nerves or rebuild neural network functions. Therefore, it is important to identify and explore feasible and effective treatment methods for relieving cancer pain.

In recent years, with the continuous exploration of methods and strategies for the treatment of tumors and cancer pain, cell therapy has been introduced to transplant functionally active cells into the host to play a pharmacological role in the treatment. Some progress has been made in pain relief by transplanting different types of cells (such as Schwann cells (Zhang et al., 2018c), olfactory ensheathing cells (Zheng et al., 2017), and neural stem cells (Lee et al., 2016) *in vivo*. A study shows that transplantation of microencapsulated Schwann cells into injured sciatic nerves can reduce the expression of P2X3 receptors in the dorsal root ganglion and relieve abnormal mechanical pain in rats (Zhang et al., 2018d). Our team previously studied the transplantation of microencapsulated neural stem cells (with the potential for self-renewal and differentiation similar to MSCs) for sciatic nerve injuries. The results showed that microencapsulated neural stem cells could significantly reduce the expression of P2X receptors, inhibit demyelination, and relieve pain (Zhang et al., 2019a; Zhang et al., 2021). It has also been found that the effect of microencapsulated neural stem cells on pain relief is better than that of non-microencapsulated neural stem cells, which may be that microencapsulation provides a role of immune isolation and protects the viability of transplanted cells.

Accordingly, MSCs have been used to treat pain (Bryk et al., 2022; Pang et al., 2014). After intravenous and intrathecal transplantation of bone marrow MSCs, they can be observed on the surface of the spinal cord and dorsal root ganglion, which can significantly relieve neuropathic pain (Liu et al., 2017). Throughout the experiment, animals showed no signs of toxicity (Liu et al., 2017). On the third day after spinal cord injury, bone marrow mesenchymal stem cell transplantation improved motor function and reduced mechanical and thermal hypersensitivity caused by spinal cord injury. Pain was improved by inhibiting the expression of protein kinase C-γ and phosphate cyclic AMP response element binding proteins in dorsal horn neurons (Watanabe et al., 2015). Bone marrow mesenchymal stem cell transplantation prevents the recruitment of blood-derived macrophages and the levels of inflammatory cytokines by restoring the blood-spinal cord barrier, protecting neurons, and relieving pain (Watanabe et al., 2015). These studies revealed that MSCs can be used in prospective cell therapies for pain treatment.

However, there is a lack of research on MSCs in cancer pain, and few reports have described their role in the treatment of cancer pain induced by chemotherapy (Al-Massri et al., 2020). Peripheral neuropathy caused by chemotherapy is one of the most common adverse reactions in cancer treatment. It often lasts for a long time after treatment and adversely affects the quality of life of patients. After cisplatin or paclitaxel treatment, nasal administration of MSCs completely reversed the signs of peripheral neuropathy caused by established chemotherapy, including mechanical hypersensitivity, spontaneous pain, and loss of nerve fibers in the epidermis of the claw (Boukelmoune et al., 2021). Intranasally administered MSCs quickly enter the meninges, spinal cord, and surrounding lymph nodes to promote IL-10 production by macrophages. The recovery of nerve fibers in the epidermis of the claw and of DRG mitochondrial function depends on the production of IL-10 (Boukelmoune et al., 2021). Interestingly, MSCs in IL-10 knockout animals could not reverse pain symptoms induced by chemotherapy (Boukelmoune et al., 2021). In another study, adipose MSCs were transplanted in a rat model of oxaliplatin-induced neuropathy. A single intravenous injection of MSCs reduced oxaliplatin-dependent mechanical hypersensitivity and relieved pain. It took effect 1 h after administration, peaked at 6 h, and lasted for 5 days (Di Cesare Mannelli et al., 2018). Adipose MSCs can reduce the oxaliplatin-induced expression of vascular endothelial growth factor and VEGF165b in the spinal cord and relieve pain (Di Cesare Mannelli et al., 2018). These studies have revealed the therapeutic role of MSCs transplantation in cancer chemotherapy-induced pain. Studies have found that MSCs decrease the levels of IL-1β and IL-6 proinflammatory cytokines, increase the secretion of leucine enkephalin (EK), and effectively relieve the pain caused by bone cancer in rats (Sun et al., 2017b). Other studies have found that MIR-9-5p modified BMSCs decreases the expression of cytokines (TNF-α and IL-6) in the spinal cord of mice by inhibiting the expression of REST gene, thus relieving cancer pain in sarcoma-inoculated mice (Zhu C. et al., 2020) (Table 1). It has been suggested that miR-9-5p gene modified BMSCs can relieve bone cancer pain by regulating inflammatory responses in the central nervous system.

**TABLE 1:** Application of MSCs in the treatment of cancer pain.

Cell source	Animal model	Animal	Mode of administration	Dosage	Biological function	Refs
Extracellular vesicles of human umbilical cord mesenchymal stem cells	Paclitaxel-induced pain model	C57BL/6J mice	i.p	(5 mg/kg) body weight of mice, twice a week for 6 weeks	Significantly reduce mitochondrial dysfunction in dorsal root ganglion and spinal cord homogenate, and reduce mechanical and thermal allergy	Kalvala et al. (2023)
Human bone marrow mesenchymal stem cells	Bone cancer pain model	Rat	Intrathecal injection	6×10^6^ cell suspension	Decrease the levels of IL-1β and IL-6 proinflammatory cytokines, increase the secretion of leucine enkephalin (EK), and effectively relieve the pain caused by bone cancer in rats	Sun et al. (2017b)
Mouse bone marrow mesenchymal stem cells	Bone cancer pain model	C3H/HeN adult mice	Infused into the spinal cord	6×10^6^ cells/10 μL were injected daily until 21 days after operation	MSCs relieve bone cancer pain by regulating the inflammatory response of the central nervous system	Zhu et al. (2020b)
Mouse mesenchymal stem cells	Pain model induced by cisplatin or paclitaxel	C57BL/6J mice	Injected intraperitoneally	1×10^6^ cell suspension, 2 cycles, 5 times a day	MSCs rapidly enters the meninges, spinal cord and surrounding lymph nodes to promote the production of IL-10 by macrophages. The relief of MSCs-mediated mechanical pain abnormalities and the recovery of DRG mitochondrial function depend on the production of IL-10	Boukelmoune et al. (2021)
Adipose-derived mesenchymal stem cells	Pain model induced by oxaliplatin	Sprague-Dawley rats	i.v (tail vein)	400 μL (5×10^3^ cells/μl) cell suspension was given a single dose on the 13th day	MSCs reduce the concentration of vascular endothelial growth factor (PAN) and upregulates the expression of VEGF165b in spinal cord tissue, and relieve the pain	Di Cesare Mannelli et al. (2018)

Current research on the use of MSCs for the treatment of pain induced by tumor chemotherapy is limited. However, there are few reports on the specific role of MSCs transplantation in pain caused by the tumor itself (growth, invasion, and metastasis), which may be related to its role in cancer and the mechanism of pain generation. As mentioned earlier, mesenchymal stem cells play a dual role in tumor progression. The tumor microenvironment consists of non-tumor cells, such as tumor-associated fibroblasts, immune cells, cell matrix components, and tumor cells, which constitute a microenvironment in which tumor cells can survive. MSCs can establish a complex mutual communication network with non-tumor and tumor cells in the microenvironment and interact to produce different effects. However, the specific mechanism underlying the interaction between stem cells and tumor cells is unclear. MSCs provide a framework for anchoring tumor cells in the form of a tumor matrix and can secrete factors that promote tumor growth. They have a strong tendency towards tumors and guide tumor homing. Therefore, the use of MSCs in cancer pain management may promote tumor progression and exacerbate cancer pain. Transplantation of MSCs into a host may promote tumor growth and metastasis by changing the microenvironment of tumor cells and biological characteristics of tumor cells. Tumor progression may further aggravate or induce nerve invasion or nerve compression, which may, in turn, aggravate refractory pain in patients with cancer. This has led to major obstacles in the current preclinical and clinical trials of MSCs that induce pain during tumor growth and metastasis. Therefore, we need to better understand the inhibitory role of MSCs in tumor progression and explore the role of MSCs as a guide for tumor treatment. For example, gene transfection methods can be used to knockdown pro-tumor growth factors, cytokines, and vascular growth factors secreted by MSCs to induce MSCs to inhibit tumor growth, which may play a therapeutic role in cancer pain. Moreover, anti-tumor immune escape can be generated by regulating the immunity between MSCs and the tumor microenvironment, for example, by changing the polarization state of macrophages. Furthermore, MSC heterogeneity may have different therapeutic effects on cancer pain. MSCs have both pro-inflammatory and anti-inflammatory phenotypes. The pro-inflammatory phenotype increases tumor growth, whereas the anti-inflammatory phenotype inhibits tumor growth. Therefore, the control of its phenotype (MSCS2) inhibits tumor growth, destroys nerve tissue, inhibits its MSCS1 type, and exerts a pain-relieving effect. Phenotypic changes in MSCs can be regulated through genetic modifications and the induction period changes to an anti-inflammatory phenotype, which may produce analgesic therapeutic effects.

MSC can have different effects on tumor progression, which may also affect cancer pain treatment. High doses of MSCs have toxic effects on tumor cells, whereas low doses of MSCs may play a promoting role. However, no relevant literature clearly reports the dose of MSCs that can produce toxic effects. Therefore, the dose of MSCs in analgesic therapy is an important factor that hinders clinical development and exploration.

MSCs can secrete neurotrophic factors (such as BDNF and NGF). Although these neurotrophic factors promote nerve injury repair and regeneration, they can also serve as pain-related factors that aggravate pain. Therefore, neural factors that may induce increased pain can be knocked out through gene transfection methods, and the positive direction of MSCs in the treatment of cancer-related pain can be fully understood. Moreover, during *in vitro* expansion of MSCs, genomic instability may accumulate, increasing the possibility of malignant transformation. Through stable regulation *in vitro*, such as using pluripotent stem cells to induce MSCs or gene reprogramming to stabilize expression, the possibility of this side effect can be reduced to better play a role in cancer pain treatment applications. Furthermore, there is insufficient evidence regarding the possible side effects and safety of MSCs transplantation and these issues require further research to explore and resolve.

Although the use of MSCs in the treatment of cancer pain is still in the primary exploration stage, many problems need to be solved, especially in clinical development, which requires more in-depth research. However, the value of mesenchymal stem cells in pain treatment cannot be denied. MSCs have several advantages including low immunogenicity, which reduces immune rejection in transplant hosts. Transplanted MSCs can repair damaged or stimulated nerves, restore nerve function, and exert analgesic effects. Direct evidence of MSCs in other types of pain shows their strong analgesic effect, and exciting results have been achieved in the application of tumor chemotherapy-induced pain. However, their transplantation still lacks direct evidence for treating cancer itself, which is closely related to the many problems that need to be solved in current MSCs transplantation techniques. In short, as researchers continue to explore, these existing problems will eventually be solved and MSCs will have broad prospects for the treatment of cancer pain.

## 7 Conclusion

Tumorigenesis and its progression can invade and destroy the nerves, leading to pain. Treatment of tumors (radiotherapy and chemotherapy) causes neuropathy, induces pain, and causes great psychosomatic damage. Increasing attention has been paid to the use of cells as a potential treatment strategy for pain. MSCs have the characteristics of self-renewal, differentiation, and low immunogenicity, which is a great prospect for cell therapy. MSCs can repair injured nerves and restore nerve function through neuroprotection, neuronutrition, immunomodulation, promotion of axonal regeneration and myelination, and inhibition of glial cell activity, which lays the foundation for pain treatment. Moreover, MSCs reduce the expression of pain-related factors (P2X, P2Y, TLR4, and NGF) and exert analgesic effects. In addition, MSCs can regulate tumor progression, which is a key factor in the treatment of cancer-related pain. These factors indicate the possibility of applying MSCs in cancer pain treatment, including tumor therapy. Although MSCs play a pharmacological role in analgesia, their bilateral function in tumors and some pain-related factors secreted by MSCs are worthy of attention. Therefore, controlling MSCs to play an active role in analgesia should solve the existing problems. Nonetheless, cell transplantation as a therapy for cancer-related pain has a long way to go.
